# Datasets associated with the characterization of produced water and Pecos River water in the Permian Basin, the United States

**DOI:** 10.1016/j.dib.2022.108443

**Published:** 2022-07-04

**Authors:** Wenbin Jiang, Xuesong Xu, Ryan Hall, Yanyan Zhang, Kenneth C. Carroll, Frank Ramos, Mark A. Engle, Lu Lin, Huiyao Wang, Matthias Sayer, Pei Xu

**Affiliations:** a Department of Civil Engineering, New Mexico State University, Las Cruces, NM 88003, United States; b NGL Partners LP, Santa Fe, NM 87501, United States; c Department of Plant and Environmental Science, New Mexico State University, Las Cruces, NM, United States; d Department of Geological Sciences, New Mexico State University, Las Cruces, NM 88003, United States; e Department of Earth, Environmental and Resource Sciences, The University of Texas at El Paso, El Paso, TX 79968, United States

**Keywords:** Water quality, Produced water characterization, Permian basin, Pecos river, Water reuse

## Abstract

The data in this report are associated with “Characterization of Produced Water and Surrounding Surface Water in the Permian Basin, the United States” (Jiang et al. 2022) and include raw data on produced water (PW) quality and Pecos River water quality in the Permian Basin, which is one of the major oil and gas producing areas in the U.S. The data include 46 samples for PW and 10 samples for Pecos River water. The data include wet chemistry, mineral salts, metals, oil and grease, volatile and semi-volatile organic compounds, radionuclides, ammonia, hydraulic fracturing additives, and per- and polyfluoroalkyl substances. The PW samples were collected from five different locations in the Permian Basin. Twenty-four of the PW samples and the ten Pecos River samples were analyzed by the authors. The information for the rest of PW samples (22 samples) was provided by industrial collaborators in the Permian Basin. Statistical analyses were performed on the combined data to obtain Mean, Max, Min, 25th percentile, 50th percentile, and 75th percentile of each analyte.

## Specifications Table

**Table:** 

Subject	Water Science and Technology
Specific subject area	Produced water (PW) quality and surrounding river water quality related to unconventional oil and gas production
Type of data	Table
How the data were acquired	To identify the chemicals of interest (COIs), the authors reviewed current PW management practices with regulations and reuse scenarios in the major production areas in the U.S. up to 2021. The investigated production areas include the Appalachian Basin (Marcellus and Utica shale areas of Pennsylvania, Ohio, and West Virginia), Oklahoma, Texas, California, Colorado, Wyoming, and New Mexico. The investigated beneficial reuse options include on-site reuse in the O&G field, irrigation, wildlife and livestock drinking water, discharge to surface water, groundwater recharge, road spreading, and land applications. Information of treated PW reuse in the O&G field was collected from the Pennsylvania case study [2]. Information of PW reuse for irrigation was derived from studies conducted in California [3], [4], [5], Colorado [6], [7], [8], and Texas [9]. Information of PW discharge to surface water and reuse for wildlife and livestock drinking water was from Texas [9], Colorado [10], and Wyoming [11]. Information of PW reuse for road spreading and land application was from Colorado [12] and Wyoming [13]. To ensure the thoroughness of the COIs list, it is compared to the United States Environmental Protection Agency (USEPA) drinking water standards, New Mexico groundwater standards [14], and New Mexico surface water standards [15]. The list of COIs was further reviewed by the New Mexico Produced Water Research Consortium. Gravimetric methods were used to measure total dissolved solids (TDS) and total suspended solids (TSS) concentrations. A Total Organic Carbon Analyzer (TOC-Vcsh, Shimadzu, Japan) was used to measure the concentrations of total organic carbon (TOC) and dissolved organic carbon (DOC). A benchtop multi-parameter meter (pH/con 300 Meter, Oakton Instruments, IL, USA) was used to measure pH. Hach COD test kits and alkalinity test kits (Hach, CO, USA) were used to measure chemical oxygen demand (COD) and alkalinity, respectively. A spectrophotometer (Hach DR6000, Hach, CO, USA) was used to measure ammonia. Ion chromatography (IC; Dionex ICS-2100, Thermo Fisher Scientific, CA, USA) was used to measure major ions. Inductively coupled plasma optical emission spectroscopy (ICP-OES; Optima 4300 DV, PerkinElmer, MA, USA) and inductively coupled plasma mass spectroscopy (ICP-MS; Elan DRC-e, PerkinElmer, MA, USA) were used to measure metals and trace elements. An alpha spectroscopy system (Mirion Technologies, Inc.) was used for the total alpha spectra acquisition and analysis. The system was equipped with 72 Passivated Implanted Planar Silicon detector which is connected to an Apex-Alpha software system [16]. A gamma spectroscopy with a broad Energy Germanium detector (BeGe, Mirion Technologies, Inc.) was used to measure radium activities [16]. A spectrofluorometer (Aqualog-UV-800-C, Horiba Instruments, NJ, USA) was used to semi-quantitatively identify the organic compositions of PW with fluorescence Emission and Excitation Matrix (FEEM). A gas chromatography (GC, Agilent 6890) coupled with a quadrupole mass spectrometry (MS, Agilent 5973) was used to measure volatile organic compounds (VOCs) and semi-VOCs. A GC (Agilent 5890) coupled with a flame ionization detector was used to measure total petroleum hydrocarbons (TPH) and organic acids. A GC (Agilent 5890) coupled with an electron capture detector was used
	to measure pesticides/herbicides. High performance liquid chromatography-tandem mass spectrometry (HPLC/MS/MS, SCIEX 5500) was used to measure per- and polyfluoroalkyl substances (PFAS). A high-resolution GC/MS (Thermo DFS) was used to measure Dioxins.
Data format	Raw Analyzed
Description of data collection	Samples for wet chemistry and inorganic analyses were collected in sterile plastic bottles. Samples for organic analyses were collected in method-specific bottles. All samples were stored at 4°C after collection and transported under chain of custody. All sample preparation and analyses followed the USEPA guidance and standard practices.
Data source location	Institution: New Mexico State University Region: Permian Basin Country: United States
Data accessibility	Repository name: Mendeley Data Data identification number: DOI: 10.17632/pfrxnjd7mw.4 Direct URL to data: https://data.mendeley.com/datasets/pfrxnjd7mw/4
Related research article	https://doi.org/10.1016/j.jhazmat.2022.128409

## Value of the Data

• These data provide the quality information of 46 PW samples and 10 Pecos River samples from the Permian Basin in New Mexico and Texas. These data are valuable for a better understanding of PW and surface water quality in the Permian Basin.
• Beneficiaries of these data include researchers, engineers, oil and gas industry, water resource managers and regulators, and other stakeholders interested in PW and surface water quality.
• These data provide the first step for risk-based assessment and designing optimal methods for treatment and potentially beneficial use of treated PW within and outside the oil and gas industry in the Permian Basin.

## 1 Data Description

**Fig. 1:**
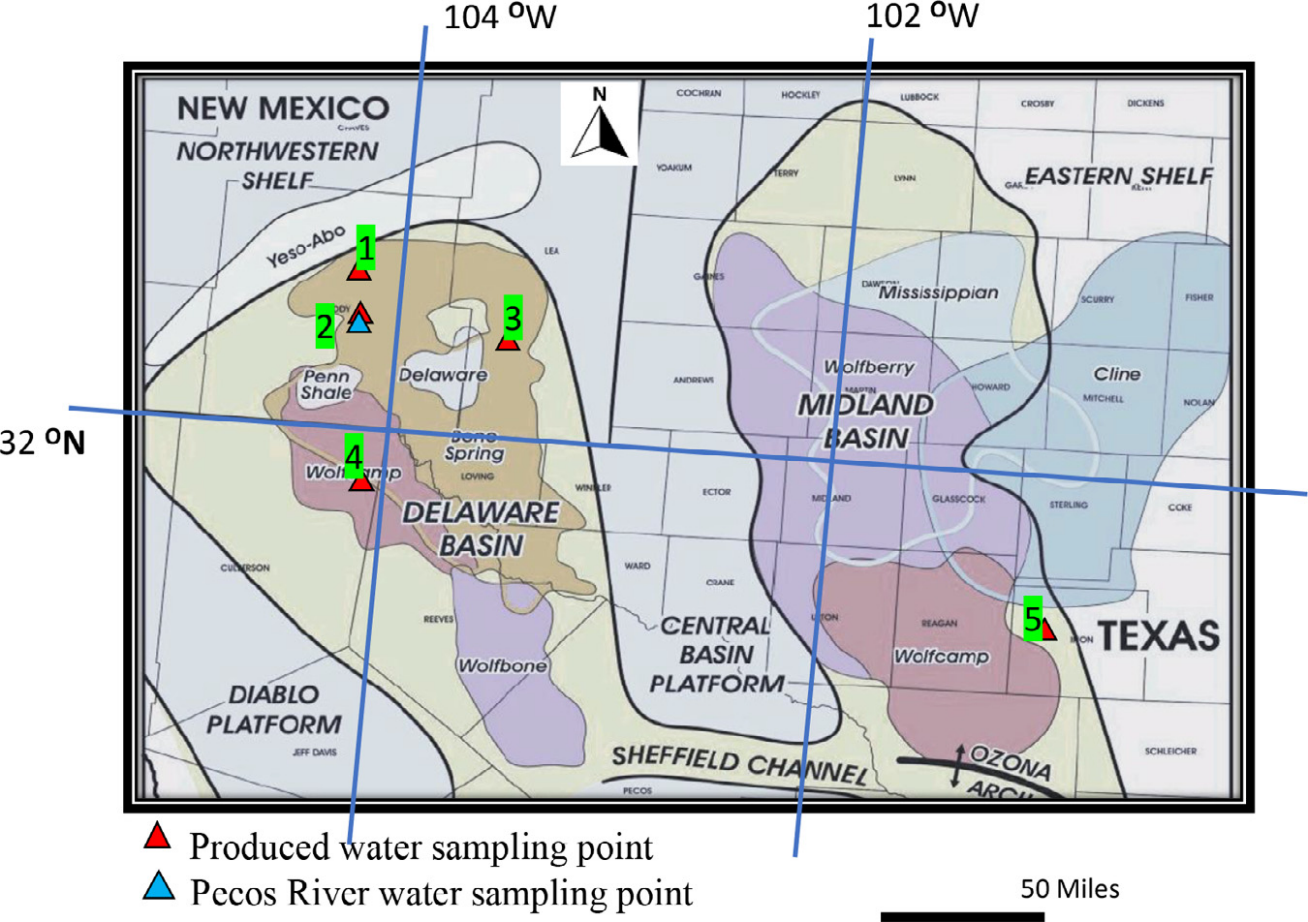
Sampling points of produced water and Pecos River water [1]. Permian Basin County map is cited from [17]. Sampling points coordinates (estimated), Point 1: 32.83, -104.3; Point 2: 32.31, -104.0; Point 3: 32.10, -103.2; Point 4: 31.49, -103.7; Point 5: 31.32, -101.1.

The data in “Produced water and Pecos River quality.xlsx” include five tabs:

• “1 Produced Water samples (PW)” includes the concentrations of 91 detected analytes in the 46 PW samples and the statistical results (Mean, Max, Min, 25th percentile, 50th percentile, and 75th percentile).
• “2 Compounds not detected in produced water (PW)” includes the names of 218 analytes that were not detected in the PW samples.
• “3 Pecos River sample (RW)” includes the concentrations of 67 detected analytes in the ten Pecos River water samples and the statistical results (Mean, Max, Min, 25th percentile, 50th percentile, and 75th percentile).
• “4 Compounds not detected in Pecos River (RW)” includes the names of 242 analytes that were not detected in the Pecos River water samples.
• “5 PW Sampling points” includes the information of PW samples based on the sampling point in Fig. 1.
• “6 RL and MDL” includes reporting limit (RL) and minimal detection limit (MDL) for the analytes in this study.

## 2 Experimental Design, Materials and Methods

TDS and TSS were measured at the same time based on EPA method 2540C and 2540 D, respectively. A water sample was first filtered through a weighed 0.15 µm glass fiber filter. The filter was dried to a constant weight at 103 to 105 °C; TSS was then measured by the increased filter weight. The filtrate was dried to a constant weight at 180 °C in a weighed dish; the dish weight increase was used to calculate TDS concentration. TOC and DOC analyses were based on EPA method 415.3. HCl was used to acidify the samples to pH < 2 to remove the inorganic carbon (carbonate and bicarbonate). Then the samples were analyzed by a TOC analyzer. Samples were filtered by a 0.45 µm filter for DOC analysis based on the procedure requirement. Alkalinity, COD, and ammonia were analyzed by following the protocols that come with the Hach test kits, respectively. Radium-226 was determined directly by measuring the 186.2 keV gamma photopeak (3.28% abundance). Radium-228 was determined indirectly by measuring Actinium-228 (t_1/2_ = 6.1 h) using 911 and 969 keV gamma photo peaks [16]. EPA method 900.0. was used for Gross Alpha and Gross Beta counts.

Organic analyses were performed by a commercial lab, Eurofins Test America. Unfiltered samples were used for the analyses. VOCs and SVOCs were measured based on EPA method 8260C and 8270D, respectively. TPH and organic acid were analyzed based on EPA method 8015D. Pesticide/herbicides were analyzed based on EPA method 8081B. PAFS were analyzed based on a modified EPA method 537. Dioxins were analyzed based on EPA method 1613B. Quantification was performed by using blank samples and external/internal standard calibration. Isotopic dilution was used for PFAS and dioxin quantitation.

The results for each sample were calculated for charge balance and mass balance. Then all the water samples were compiled together for statistical analysis. Statistical analyses were performed by using Excel to get Mean, Max, Min, 25% percentile, 50% percentile, and 75% percentile values for each analyte.

## Ethics Statements

This study followed the ethical guidelines that all authors must comply with.

## CRediT authorship contribution statement

**Wenbin Jiang:** Methodology, Investigation, Formal analysis, Writing – original draft, Writing – review & editing. **Xuesong Xu:** Methodology, Investigation, Formal analysis, Writing – original draft, Writing – review & editing. **Ryan Hall:** Methodology, Investigation, Formal analysis, Writing – original draft, Writing – review & editing, Supervision, Funding acquisition. **Yanyan Zhang:** Writing – review & editing, Supervision, Funding acquisition. **Kenneth C. Carroll:** Writing – review & editing, Supervision, Funding acquisition. **Frank Ramos:** Methodology, Investigation, Formal analysis, Writing – original draft, Writing – review & editing. **Mark A. Engle:** Methodology, Investigation, Formal analysis, Writing – original draft, Writing – review & editing. **Lu Lin:** Methodology, Investigation, Formal analysis, Writing – original draft, Writing – review & editing. **Huiyao Wang:** Writing – review & editing, Supervision, Funding acquisition. **Matthias Sayer:** Writing – review & editing, Supervision, Funding acquisition. **Pei Xu:** Methodology, Investigation, Formal analysis, Writing – original draft, Writing – review & editing, Supervision, Funding acquisition.

## Declaration of Competing Interest

The authors declare that they have no known competing financial interests or personal relationships that could have appeared to influence the work reported in this paper.

The authors declare the following financial interests/personal relationships which may be considered as potential competing interests.

## Data Availability

Datasets associated with the characterization of produced water and Pecos River water in the Permian Basin (Original data) (Mendeley data).
